# An Optical Planar Waveguide-Based Immunosensors for Determination of *Fusarium* Mycotoxin Zearalenone

**DOI:** 10.3390/toxins13020089

**Published:** 2021-01-25

**Authors:** Alexei Nabok, Ali Madlool Al-Jawdah, Borbála Gémes, Eszter Takács, András Székács

**Affiliations:** 1 Materials and Engineering Research Institute, Sheffield Hallam University, Howard Street, Sheffield S1 1WB, UK; aalimadlol@yahoo.com; 2 College of Sciences, Babylon University, P.O. Box 4, Hilla 51002, Iraq; 3 Agro-Environmental Research Institute, National Research and Innovation Centre, Herman Ottó út 15, H-1022 Budapest, Hungary; szekacs.andras@akk.naik.hu (A.S.); gemes.borbala.leticia@akk.naik.hu (B.G.); akacs.eszter@akk.naik.hu (E.T.)

**Keywords:** mycotoxin, zearalenone, planar waveguide sensor, polarisation interferometer, label-free detection, limit of detection

## Abstract

A planar waveguide (PW) immunosensor working as a polarisation interferometer was developed for the detection of mycotoxin zearalenone (ZON). The main element of the sensor is an optical waveguide consisting of a thin silicon nitride layer between two thicker silicon dioxide layers. A combination of a narrow waveguiding core made by photolithography with an advanced optical set-up providing a coupling of circular polarised light into the PW via its slanted edge allowed the realization of a novel sensing principle by detection of the phase shift between the p- and s-components of polarised light propagating through the PW. As the p-component is sensitive to refractive index changes at the waveguide interface, molecular events between the sensor surface and the contacting sample solution can be detected. To detect ZON concentrations in the sample solution, ZON-specific antibodies were immobilised on the waveguide via an electrostatically deposited polyelectrolyte layer, and protein A was adsorbed on it. Refractive index changes on the surface due to the binding of ZON molecules to the anchored antibodies were detected in a concentration-dependent manner up to 1000 ng/mL of ZON, allowing a limit of detection of 0.01 ng/mL. Structurally unrelated mycotoxins such as aflatoxin B1 or ochratoxin A did not exert observable cross-reactivity.

## 1. Introduction

Label-free optical biosensor techniques based on evanescent field effects are of increasing interest for agro-environmental safety in monitoring the quality of food and animal feed [1]. Evanescent waves (or fields) created at interfaces between two transparent media having different refractive indices (RIs) in optical devices such as waveguides propagate along the interfaces with their intensity decaying rapidly away from the interfaces. The evanescent field can penetrate into the medium of the lower RI to a distance of approximately 200 nm [2], indicating that the evanescence phenomenon is sensitive to RI changes not only in the waveguide but also in its immediate vicinity, and therefore can undergo characteristic modulations due to the changes in the medium RI caused by molecular interactions taking place near that surface. While these interactions can be detected in real time and with outstandingly high sensitivity, the use of molecular size bio-receptors (antibodies, aptamers, or molecularly imprinted polymers) in the biosensor set-up introduces the required specificity to the target analytes for analytical determination. Several waveguide-based biosensor formats exist for the optical detection of various mycotoxin molecules such as zearalenone (ZON).

Label-free optical immunosensor techniques include reflectance-based methods, such as total internal reflection ellipsometry (TIRE) [3], and grating-based methods, such as optical waveguide light-mode spectroscopy (OWLS) [4]. However, these methods typically rely on lab-based equipment and as a consequence are often incapable of fulfilling the current demands of portable biosensors suitable for in-field analysis, as the laboratory benchtop instrument cannot be moved to and operated at the site of sampling. One of the most promising directions in label-free biosensing is based on the use of the most sensitive optical technique of interferometry. Several successful developments of interferometric biosensor devices were accomplished recently [5]; they include dual beam interferometers [6], ring-resonators [7], and Mach-Zehnder (MZ) interferometers [6,8]. Biosensors based on MZ interferometers combining a high sensitivity of detection and portable design [6,7,8,9,10] were the most impressive with a pinnacle achievement being a monolithic silicon-based MZ biosensor combining in one chip the light source, multichannel biosensor with microfluidic sample delivery, photodetectors, and signal processing electronics [11,12]. Such biosensors are particularly suitable for in-field or point-of-need use. Thus, optical immunosensors based on planar waveguide (PW) technology are gaining attention [12] and have been developed for mycotoxins such as deoxynivalenol, ochratoxin A, ZON, and T-2 [13,14,15,16,17] and other aquatic toxins including sweet water and marine algal toxins such as microcystins, okadaic acid, domoic acid, and cylindrospermopsin involved in direct toxicity [18,19,20,21], saxitoxin involved in indirect toxicity through the food chain [22], as well as a microbial toxin tetrodotoxin [23]. In the current work, a PW immunosensor based on a novel principle of polarisation interferometry was adapted for the detection of ZON.

## 2. Results and Discussion

### 2.1. Planar Waveguide Biosensor Design and Testing

The detection principle of the planar waveguide (PW) biosensor acting as a polarisation interferometer (PI) is similar to Mach–Zehnder (MZ) interferometers, but instead of two optical arms in the MZ biosensor, the p- and s-polarisations of light were used as parallel parameters in this set-up. The PW being the main element of the developed biosensor was devised on an Si wafer and consisted of a 200 nm thick silicon nitride (Si_3_N_4_) ore layer (having a RI *n* = 2.01) placed between two much thicker (3 µm) cladding silicon oxide (SiO_2_) layers of lower RI (*n* = 1.46). Such design allowed the propagation of a single mode electromagnetic (EM) wave through the waveguide by multiple internal reflection; the large difference in RIs between the Si_3_N_4_ core and the SiO_2_ cladding resulted in light propagation at a steep angle of 47°, creating a large number of internal reflections of light (up to 3000 reflections/mm) along the PW.

**Figure 1:**
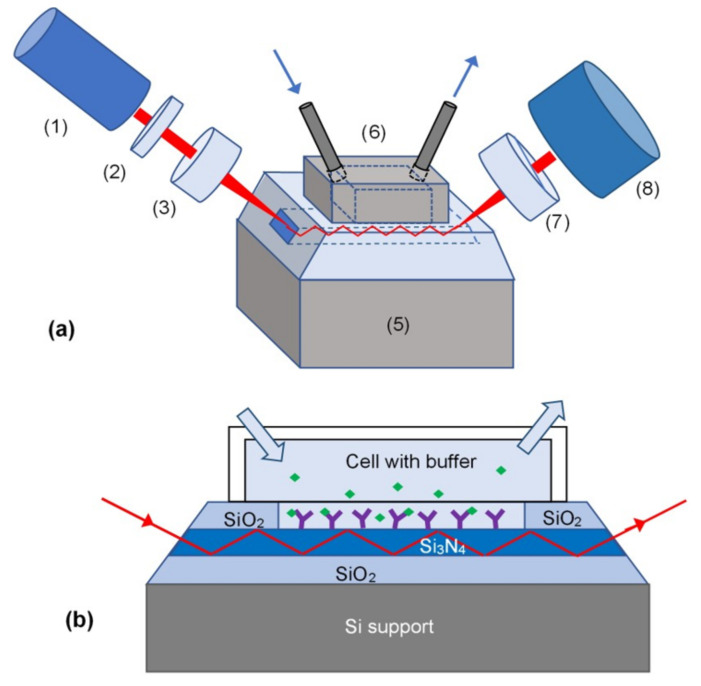
(**a**) The planar waveguide (PW) biosensor experimental set-up: laser diode (1), λ/4 plate (2), collimating lens (3), PW (4) on Si wafer support (5), reaction cell (6) with inlet and outlet tubes, polariser (7), and charge-coupled device (CCD) array (8); (**b**) Cross-section of waveguide section showing schematically the multiple reflections of light, the sensing window, the reaction cell, and antibodies immobilised on the PW surface binding zearalenone molecules.

As shown in Figure 1, the polarised 630 nm light from a laser diode (1) was coupled into waveguide (4) via a slant edge, which was polished at a 47° angle to provide a 90° incidence angle and therefore maximal efficiency of coupling. The light was converted to circular polarisation using a λ/4 plate (2) and focused on the slant edge using a lens (3). The outcoming light is going through a polariser (7), which converts the changes in the EM wave polarisation into modulation of its intensity, and collected by a charge-coupled device (CCD) array photodetector (8). The waveguide (4) with the dimensions of 25 × 8 mm is held between two pieces of black nylon with the upper piece forming an 8 × 2 × 6 mm (≈0.1 mL) cell (6) sealed against the top side of the waveguide and equipped with inlet and outlet tubes enabling injecting different chemicals into the cell. In the earlier versions of the set-up, the top layer of SiO_2_ is etched away by injecting 1:10 diluted hydrofluoric acid into the cell to form the sensing window. Later on, in the advanced experimental set-up, both the waveguiding core and sensing window were formed by photolithography.

The resulted set-up operates as a planar polarisation interferometer (PPI); the p-component of polarised light (lying in the plane of incidence) is affected by changes in the RI of the medium, while the s-polarised component (orthogonal to the plane of incidence) is almost invariant to the RI variation in the medium and subsequently used as a reference. Any changes in the medium RI in the sensing window including the variations of RI caused by molecular adsorption result in a multi-periodic sensor response cause by a variable phase shift between p- and s- polarisations of light, which could be converted by a polariser to a multiperiodic signal. In a way, the principle of PI is a logical expansion of the TIRE method [3], which is based on the detection of a phase shift between p- and s-components of polarised light, utilising a large number of reflections in the optical waveguides.

**Figure 2:**
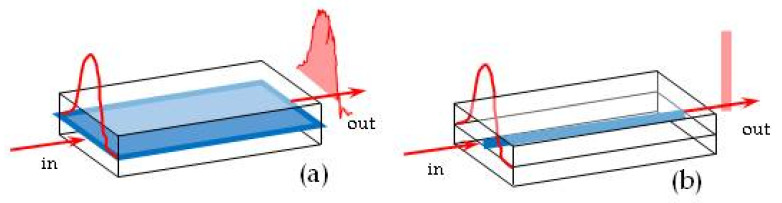
Propagation of light through the planar waveguide (PW) in a wide core (**a**) and narrow core (**b**) set-up.

**Figure 3:**
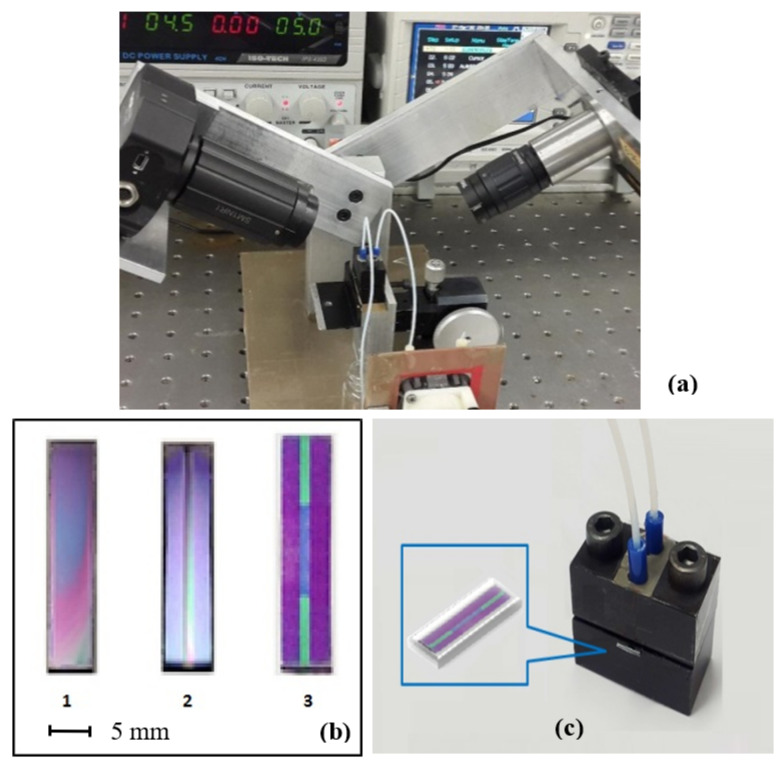
The upgraded planar polarisation interferometry device. Planar waveguide-based polarisation interferometry experimental set-up upgraded from the prototype (**a**); photolithography steps the waveguide preparation (**b**); the reaction cell with the waveguide inserted (**c**).

The experimental set-up for PI went through several stages of optimisation. Previously, the light from a fan-beam laser diode was coupled into the PW and was propagated over the entire width (≈8 mm) of the waveguide. As the result of a modal dispersion of light across the waveguide, and therefore not equal conditions of light propagation (see Figure 2a), averaging of the light intensity over the entire width of the waveguide has led to losing the contrast of the interference pattern. To avoid that, the number of pixels for light averaging had to be limited. However, improved results were obtained with photolithography to form a narrow strip (≈2 mm) of silicon nitride (Figure 2b). Another advantage of photolithography was the formation of a well-defined sensing window. The photographs of Figure 3 show the PW biosensor set-up (Figure 3a), the top views of the waveguide at different preparative stages in Figure 3b, e.g., a 24 × 6 mm chip with an SiO_2_ and Si_3_N_4_ layer deposited (1), after etching of Si_3_N_4_ to form a narrow (2 mm) waveguide core (2), and the final structure with the sensing window (2 × 8 mm) etched in the top SiO_2_ layer (3), and the waveguide inserted in the cell (Figure 3c). A Thorlabs LC100 camera was interfaced to a PC; the output signal acquisition was carried out using SPLICCO software (A Thorlabs GmbH, Bergkirchen, Germany).

### 2.2. Testing the Waveguide Sensor

The testing of a PW sensor was performed by varying the RI of the liquid medium, i.e., by injecting into the cell initially filled with water aqueous solutions of NaCl of different concentrations having different RIs and recording the corresponding multi-periodic output waveforms [15,16]. The number of periods of signal oscillation were roughly estimated from these waveforms and presented in Table 1 along with corresponding changes in the RI, which allows estimating the refractive index sensitivity (RIS) of the PW sensors as:

$$RIS=\frac{2\times \pi \times N}{\Delta n}$$

where *N* is the number of periods of oscillation, and Δn is the difference between the RI of aqueous sodium chloride (NaCl) and water i.e., $\Delta n=n_{NaCl}-n_{water}$, where $n_{water}=1.332$. The obtained RIS values are also shown in Table 1.

An average RIS around 5100 radians per RI unit was calculated and is quite remarkable, since it is much higher than in traditional optical methods, e.g., surface plasmon resonance or total internal reflection ellipsometry. Relatively large standard deviation values are due to a rough estimation of a number of periods of oscillations in the output waveforms.

### 2.3. Detection of Zearalenone by Planar Waveguide Immunoosensor

The detection of ZON was carried out using the experimental PW set-up described in Section 2.2, which has an RIS of approximately 5100 rad/RI unit. Experiments of detecting ZON were performed in a direct immunosensor format with specific antibodies immobilised electrostatically on the sensor surface via the layers poly-allylamine hydrochloride (PAH) and protein A (ProtA) in a following sequence: (i) deposition of PAH polycations carrying positive charge; (ii) deposition of ProtA being negatively charged at pH = 7, (iii) deposition of polyclonal antibodies to ZON via a biding site at the second constant domain to ProtA. In the above experiments, a very thin (≈1 nm) layer of PAH [24] deposited on the waveguide surface yields a phase shift of about a ½ period. The absorption of larger molecules of ProtA (42 kDa) causes a larger phase shift of about two periods, while much larger molecules of polyclonal antibodies to ZON (150–900 kDa) gave about 3.5 periods of phase change.

**Figure 4:**
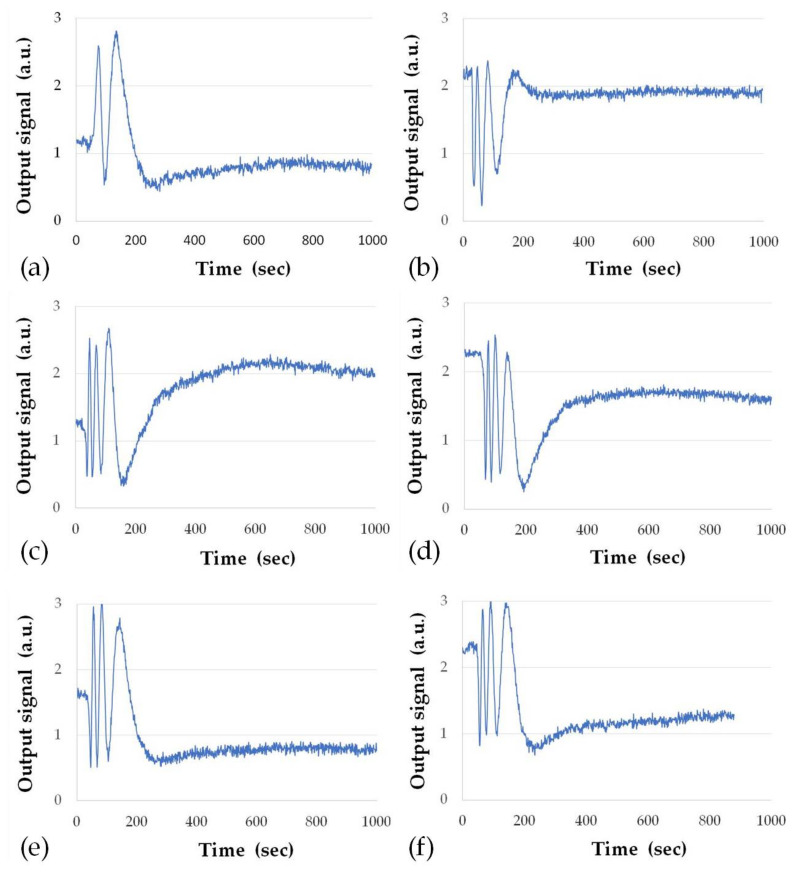
Typical sensor responses to injection of zearalenone (ZON) of different concentrations: 0.01 ng/mL (**a**), 0.1 ng/mL (**b**), 1 ng/mL (**c**), 10 ng/mL (**d**), 100 ng/mL (**e**), and 1000 (ng/mL) (**f**).

The detection of ZON was undertaken by sequential injections of ZON standards in increasing order of concentrations, e.g., 0.01, 0.1, 1, 10, 100, and 1000 ng/mL. Typical responses to injections of different concentrations of ZON are shown in Figure 4. As one can see, the number of periods of signal oscillation increases with the increase in ZON concentration. The accuracy of phase shift calculation was about 0.1 of a period or about 0.6 rad. However, the increase in concentration of ZON cannot lead to a limitless increase in the number oscillation periods of the output signal. The sequential injections of ZON at increasing concentration cause a gradual saturation of the ZON-specific antibodies. The saturation and even slight decrease of the individual phase shifts is due to the beginning saturation of the binding sites of the antibodies. A complete saturation of binding sites resulted in a very small phase shift due to non-specific binding. This corresponds to the exhaustion of the immobilised antibodies on the sensor surface. It has also to be noted that the washing out of non-specifically bound ZON molecules during purging with pure buffer solution through the cell caused a phase shift of about 1/4 of a period, which was the baseline (background) level of the experiments.

As seen in Figure 4, the minimal detection concentration of ZON (limit of detection, LOD) was 0.01 ng/mL, which is an order of magnitude lower than the results obtained earlier using TIRE in a similar direct immunoassay format and have the same LOD as in TIRE measurements in more sensitive competitive assay format [3]. Cross-reactivities (CRs) of the immobilised antibodies, which were carried out with aflatoxin B1 (AFB1) and ochratoxin A (OTA), did not show any signal oscillations and thus no phase shifts, even at a concentration of 1000 ng/mL. This demonstrated the high specificity of the antibodies towards ZON.

### 2.4. Comparison of ZON Detection with PW and ELISA Methods

**Figure 5:**
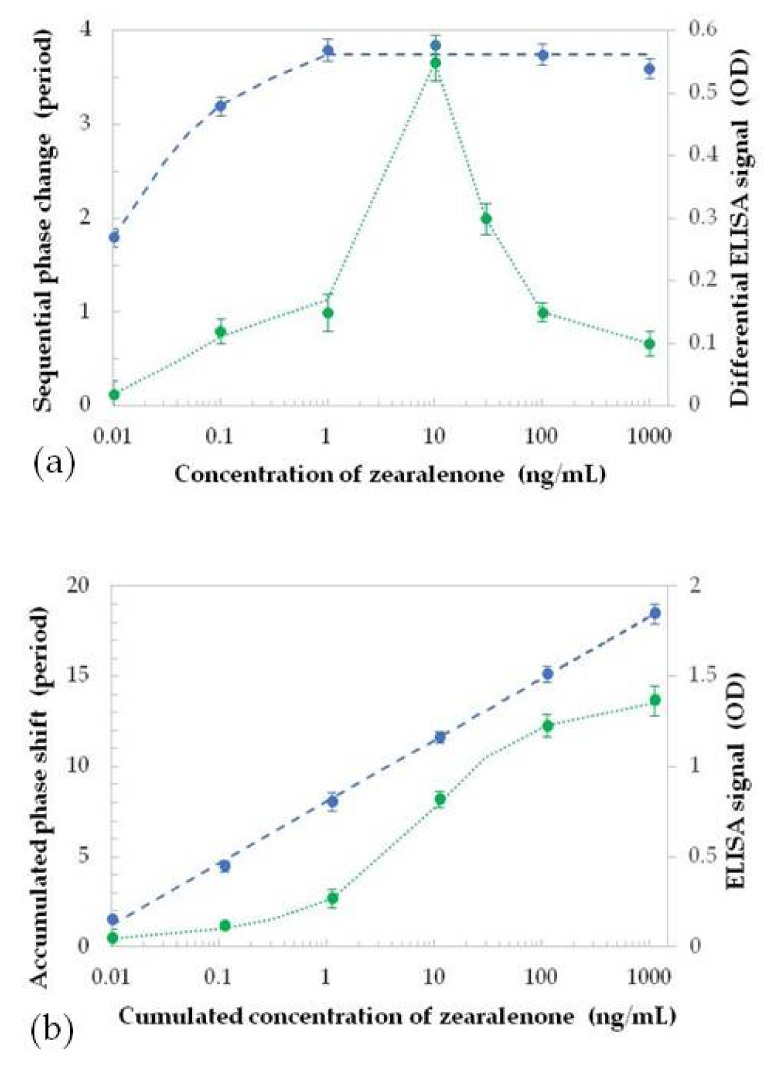
Dependence of the assay signals in the PW sensor (phase shifts, blue symbols, and blue slashed line) and the enzyme-linked immunosorbent assays (ELISA) (optical densities, green symbols, and green dotted line) caused by injections of zearalenone (ZON) in different concentrations. Total responses against the accumulated concentration of ZON (**a**); differential responses among subsequent injections (**b**).

The analysis of the enzyme-linked immunosorbent assays (ELISA) detection of ZON in the optimised direct immunoassay with respective specific antibodies and 3,3′,5,5′-tetramethylbenzidine (TMB) as a chromophore showed a saturation curve in a semi-logarithmic plot, while increments in the accumulated phase shift due to various concentrations of ZON could also be plotted. Such signal saturation and accumulated responses for the injections of ZON are given in Figure 5.

A semi-logarithmic graph (Figure 5a) indicates that the sequential number of periods of phase change initially increases with increasing ZON concentrations; then, from 1 ng/mL ZON, the response becomes constant (approximately four periods per one order of magnitude change in ZON concentration). This constant increase in the phase shift steadily continues as the concentration of ZON is increased to 1000 ng/mL. Therefore, as seen, injections of 1, 10, 100, and 1000 ng/mL of ZON, respectively, resulted in the same increase in the analytical signal (number of periods of phase change). Since the biosensing test was performed by consecutive injections of ZON, the total phase shifts for each concentration can be calculated by adding the responses of individual injections and subtracting the background of non-specific binding. Such dependence of a total phase shift against the accumulated concentration of ZON (Figure 5b) indicates that the sensor response is very close to linear, which means that the saturation of binding centres was not achieved. The low LOD could be estimated as 0.01 ng/mL by extrapolation of the linear dependence to the triple noise level being approximately 1.8 rad.

The results of ELISA control measurements are presented in Figure 5 for comparative assessment. As the ELISA was performed in a competitive assay format, its calibration resulted in a decreasing sigmoid curve, showing that the ratio of bound antibodies to the immobilised conjugate of ZON to bovine serum albumin (BSA) onto the surface decreases with increasing concentrations of ZON. Thus, the higher the concentration of ZON is in the sample, the lower the analytical signal becomes. For a straight comparison with the direct immunosensor format, this sigmoid standard curve was inverted by showing the ratio of the unbound antibodies, and this proportion shows an increasing trend as a function of ZON concentration with a saturation of the binding sites on Figure 5b. The differential diagram of that sigmoid standard curve, plotted in Figure 5a, shows a bell-shaped curve for the incremental signals with a maximum at 10 ng/mL and a decreasing rate of signal increases between 10 and 1000 ng/mL, indicating the saturation of the antibody binding sites available on the surfaces of the wells of the microplates. This means that near the peak of the bell-shaped curve (corresponding to the half maximal inhibitory concentration or IC_50_ value i.e., the inflection point of the sigmoid curve), the increase in the analytical signal is the highest. Towards the right and left tails (the lower and upper plateaus of the sigmoid curve), the differences in the analytical signal between the measured ZON concentrations decrease.

The actual smallest concentration detected in these experiments was 0.01 ng/mL, corresponding to 1.5 periods of phase change in the PW sensor, while approximately 0.8 ng/mL was achieved in the ELISA test. The PW sensor baseline was established by injecting pure buffer solution (with no toxin content), which gave a response of a quarter-of-a-period of phase change related to small changes in RI due to washing out non-specifically bound ZON molecules. The intercept of the baseline with the initial linear slope of PW sensor response gives a value of 0.004 ng/mL (Figure 5b). This indicates that the PW sensor is capable of detecting at least two orders of magnitude lower ZON concentrations that the corresponding competitive ELISA using the same antibodies, and it represents an outstanding sensitivity for optical biosensors operating in the direct immunoassay format. The performance of the immunoreagents in other immunoassay formats indicates proper robustness for application e.g., in surface water. Moreover, the sensitivity can be increased even further by the use of a reference channel, e.g., the section of the waveguide without immobilised antibodies.

## 3. Conclusions

The development of a PW biosensor for the detection of ZON was achieved in several steps. A prototype of a PW biosensor operating as a PI was devised using a silicon nitride waveguide layer sandwiched between two silicon oxide layers. The prototype was upgraded to a narrow core waveguide and the sensing window made by photolithography and the improved optical system, which resulted in a better quality of signal and therefore a higher refractive index sensitivity. The biosensor was characterised for its refractive index sensitivity under standardised conditions by creating spectrograms of the signal waveforms in response to injections of aqueous NaCl solutions of different concentrations. The sensor surface was functionalised using electrostatically deposited PAH and ProtA on the sensor surface to entrap ZON-specific antibodies. Immunosensing of ZON was carried out by recording the PW sensor responses caused by sequential injections of ZON solutions at increasing concentrations in the range of 0.01 and 1000 ng/mL. The application of monoclonal antibodies could provide better analyte specificity (specific recognition of ZON); therefore, it is a possible direction of further development in this biosensor approach to apply monoclonal antibodies. In this study, advantages of polyclonal antibodies were utilised, namely, that polyclonal antibodies are often of higher affinity than monoclonals. The quantification of ZON in the direct immunosensor was plotted as a saturation curve with increasing ZON concentrations, the corresponding incremental built-up of the sensor signal in relation to ZON concentrations was calculated, and the LOD of the methods was determined to be below 0.01 ng/mL.

The developed PW experimental set-up is still a benchtop type because of other electronic equipment used; the dimensions of the optical assembly is only about 10 × 20 × 20 cm, and it could be scaled down further down using miniature optical components. We are planning to develop a portable hand-held biosensor including the signal processing electronics, which will be suitable for in-field use.

## 4. Materials and Methods

### 4.1. Reagents and Instrumentation

All chemical reagents, including PAH, ProtA, mycotoxin standards, BSA, 2-amino-2-(hydroxymethyl)propane-1,3-diol (tris(hydroxymethyl)aminomethane, Tris) buffer and biochemicals were purchased from Sigma-Aldrich Co. (Dorset, UK or Budapest, HU), unless indicated otherwise. ZON-specific antibodies and protein conjugates were obtained at the Agro-Environmental Research Institute, National Research and Innovation Centre, Budapest, Hungary, as described before [4]. Immunoassays were performed in a SpectraMax iD3 Multi-Mode Microplate Reader (Molecular Devices, San Jose, CA, USA) using high-capacity 96-well microplates (Nunc, Roskilde, Denmark).

### 4.2. Planar Waveguide Immunosensor Design

The PW structures were devised by standard microelectronic processes as a thin (200 nm) layer of Si_3_N_4_ sandwiched between much thicker (3 µm) layers of SiO_2_. The 200 nm core layer thickness was required to accommodate a single mode electromagnetic wave propagating along the waveguide [25,26]. Due to the large difference in RIs between the Si_3_N_4_ core (*n* = 2.01) and the SiO_2_ cladding (*n* = 1.46), the light propagates at an angle of 47° (corresponding to an angle of total internal reflection) and with a consequent 3000 reflections/mm approximately. The use of a slant edge of the waveguide cut at 47° was an optimal light coupling solution, which provided sufficient light intensity propagating through the waveguide.

In the experimental PI set-up, 650 nm light from a laser diode was focused with a lens to a narrow (less than a millimetre) spot on a slant edge of the waveguide and collected on the other side with a CCD array. A polarising element in front of a CCD camera allows the visualisation of a phase shift between p- and s-components of polarised light. The reaction cell equipped with the inlet and outlet tubes is sealed against a sensing window, which was etched in the top SiO_2_ layer. The surface of Si_3_N_4_ in the sensing window could be coated with a biosensing layer. Any changes in RI and/or thickness of this sensing layer affect mostly the p-component of polarised light (while the s-component acts as a reference), thus resulting in a multi-periodic output signal.

### 4.3. Functionalisation of the Sensor Surface

**Figure 6:**
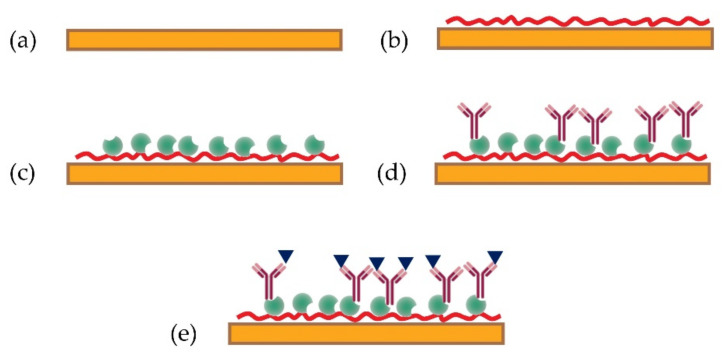
Steps in functionalisation of the sensor surface, and detection of zearalenone (ZON) with the functionalised sensor. Bare planar waveguide sensor (**a**); electrostatic immobilisation of a poly-allylamine hydrochloride (PAH) layer (**b**); binding protein A (Prot A) to PAH (**c**); oriented anchoring of ZON-specific antibodies by Prot A (**d**), detection of ZON by its specific binding to the anchored antibodies (**e**).

The immunoreagents were immobilised onto the biosensor transducer, as depicted in Figure 6, using PAH as a polycation electrostatically deposited on the biosensor surface. A thin (10–20 nm) layer of SiO_2_ was left of the surface of the Si_3_N_4_ core to provide a negative surface charge of OH^−^ for binding PAH polycations; this was achieved using ellipsometry thickness measurements to calibrate the etching time. The coating layer was deposited by incubating the sensor for 20 min with a 1 mg/mL aqueous solution of PAH. After removal of the solution, the sensor was rinsed three times with de-ionised water, and it was coated by incubating the sensor with a 0.01 mg/mL solution of ProtA in 35 mM Tris-HCl buffer (pH = 7.5) for 15 min. After being rinsed three times with Tris-HCl buffer, incubation with polyclonal antibodies to ZON at a concentration of 1 µg/mL in Tris-HCl buffer was carried out for 15 min. Finally, after final rinsing the cell three times with Tris-HCl buffer, biosensing tests were performed by injecting ZON aliquots at increasing concentrations into the cell and recording the PW sensor responses.

### 4.4. Planar Waveguide Immunosensor Assay

The detection of ZON was carried out in direct assay with specific polyclonal antibodies (Ab) immobilised electrostatically on the waveguide surface via a polycation layer of PAH followed by electrostatic binding of protA, which have a binding site to immunoglobulins (IgG or IgM) following the procedure described in detail earlier [16] (see Section 4.3). Then, the detection of ZON was carried out by sequential injections of ZON solutions with progressively increasing concentration of ZON of 0.01, 0.1, 1, 10, 100, and 1000 ng/mL in phosphate buffer. Typical sensor responses to the injection of different concentrations of ZON were recorded and evaluated. Each injection was followed by purging the cell three times with 1 mL of pure buffer solution in order to remove unbound mycotoxin molecules. Before each series of measurements, the cell was thoroughly cleaned in ethanol and de-ionised water. Negative tests were carried out by injecting structurally unrelated mycotoxins, e.g., AFB1 and OTA at concentrations up to 1000 ng/mL.

### 4.5. Enzyme-Linked Immunosorbent Assay

Enzyme-linked immunosorbent assays (ELISAs) were carried out in 96-well microplates (see Section 4.1) in a competitive immunoassay format. Microplate wells were coated with 1 µg/mL ZON–BSA conjugate in carbonate buffer (15 mM Na_2_CO_3_, 35 mM NaHCO_3_, pH = 9.6) for overnight (≈8 h) at 4 °C. The unbound conjugate was washed out 4 times with phosphate buffer saline (PBS) (137 mM NaCl, 2.7 KCl, 10 mM Na_2_HPO_4_ × 2H_2_O, pH = 7.4) with 2% Tween20. Blocking was carried out with 150 µL/well 1% gelatine in PBS for 1.5 h of incubation at 37 °C. After washing, competition was performed by adding 50 µL/well of both ZON analytical standard and purified rabbit anti-ZON serum (in PBS buffer with 5% Tween20, dilution 1:1000). After 1 h of incubation at 37 °C and four times washing, 100 µL/well goat anti-rabbit IgG-HRP (horseradish peroxidase, dilution 1:7500) conjugate were added as secondary antibody and incubated for 1 h at 37 °C. Unbound secondary antibodies were washed out 4 times with PBS, and enzymatic activity was measured using 100 µL/well substrate solution containing 1.3 mM hydrogen peroxide as a substrate and 0.42 mM TMB as a chromophore in 100 mM citrate buffer (pH = 6.0). After 10 min of incubation, the enzymatic activity was stopped by 50 µl of 4 M sulphuric acid (H_2_SO_4_), and absorbance was measured at a wavelength of 450 nm.

## Figures and Tables

**Table 1:** Evaluation of the refractive index sensitivity (RIS) of the planar waveguide sensor as a function of the number of periods of oscillation (N) and differences in the refractive index (△n) in aqueous sodium chloride (NaCl) solutions at different concentrations (NaCl%).

NaCl%	N	△n	No. of Periods	RIS (rad/RI unit)
2	1.3370	0.0050	3	3769.90
5	1.3395	0.0075	6	5026.55
8	1.3420	0.0100	10	6283.19
10	1.3460	0.0140	12.5	5610.00
15	1.3495	0.0175	16	5744.63
20	1.3610	0.0290	19	4166.57
	Average RIS = 5091.8 ± 787.5 rad/RI unit			

## Data Availability

The data presented in this study are available on request from the corresponding author. The data are not publicly available due to privacy reasons.
